# A cream of herbal mixture to improve melasma

**DOI:** 10.1111/jocd.12938

**Published:** 2019-04-13

**Authors:** Qiongyu Zhang, Ying Tu, Hua Gu, Dongjie Sun, Wenjuan Wu, Mao‐Qiang Man, Hongduo Chen, Haiyang Liu, Xiao Ma, Li He

**Affiliations:** ^1^ Department of Dermatology First Affiliated Hospital of Kunming Medical University Kunming China; ^2^ Dermatology Service Veterans Affairs Medical Center San Francisco San Francisco California; ^3^ Department of Dermatology University of California San Francisco San Francisco California; ^4^ Department of Dermatology The First Hospital of China Medical University Shenyang China; ^5^ State Key Laboratory of Phytochemistry and Plant Resources in West China Kunming Institute of Botany, Chinese Academy of Sciences Kunming China; ^6^ Beitaini Bio‐technological Co Ltd Kunming China

**Keywords:** erythema index, herbal mixture, melanin index, melasma, melasma area and severity index score

## Abstract

**Background:**

Melasma is an acquired, common hyperpigmented disorder on the face. While many therapeutic approaches are available, their efficacy is moderate.

**Objective:**

To investigate the safety and efficacy of a cream containing herbal mixture for melasma.

**Methods:**

A total of 90 volunteers with melasma were enrolled in this randomized, double‐blind, controlled clinical study, and they were randomly divided into three groups (A, B, and C). Patients in group A were treated with a cream containing herbal mixture, while groups B and C were treated with arbutin cream and placebo, respectively, twice daily for 12 weeks. Melasma area and severity index (MASI) score, melanin index (MI), erythema index (EI), changes in density of inflammatory cells, and adverse events were evaluated every 4 weeks.

**Results:**

Although MASI scores declined significantly in both groups A and B (*P* < 0.05), a greater reduction was seen in group A (13.00−9.82 = 3.18 for group A; 12.65−10.84 = 1.81 for group B). Moreover, the cream containing herbal mixture, but not arbutin cream and placebo, significantly reduced EI and density of inflammatory cells after 12‐week treatment (*P* < 0.05). No adverse reactions were observed in either group A or group C. In group B, two subjects experienced mild erythema and itching, which disappeared after stop using the arbutin cream.

**Conclusion:**

The cream containing herbal mixture is safe and effective for melasma.

## 1 INTRODUCTION

Melasma is an acquired pigmentary condition, commonly occurring on the face of females, which can be classified into centrofacial pattern, malar pattern, mandibular pattern, and mixed.^1^, ^2^, ^3^ The prevalence of melasma is 1% in general population and as high as 50% in high‐risk populations.^4^ Previous studies have shown that at least four pathogenesis are involved in the development of melasma, that is melanogenesis/melanin,^5^ inflammation,^6^, ^7^, ^8^, ^9^ vascularization/vascular factor,^10^, ^11^ and defective skin barrier.^12^, ^13^, ^14^, ^15^ Although melasma is not life‐threatening, it greatly impacts the quality of the life of patients.

Although many therapeutic regimens are available for melasma, the efficacy is moderate.^4^ In some cases, they can cause severe adverse reactions. For example, repeated applications of hydroquinone, commonly used for the treatment of hyperpigmentation, can cause toxic reactions, depigmentation, vitiligo‐like hypochromia, or leukoderma.^16^, ^17^ Therefore, development of effective and safe products for melasma is becoming emergent.

In the present study, we evaluated the efficacy and safety of a newly developed formulation in a randomized, double‐blind, and controlled trial in 90 volunteers. Certain ingredients in this formulation specifically target respective aspect involved in the pathogenesis of melasma. For example, China camellia can antioxidant and inhibit tyrosinase activity,^18^ leading to inhibition of melanogenesis. Sanchi can promote blood circulation by suppressing the aggregation of platelets.^19^ Portulaca oleracea exhibits anti‐inflammatory and anti‐allergy properties,^20^ resulting in improvement in inflammation. Prinsepia utilis can improve epidermal permeability barrier function, via stimulation of epidermal ceramide production.^21^

## 2 MATERIALS AND METHODS

### 2.1 Study design

This randomized, double‐blind, controlled clinical study was carried out during winter (2017‐2018) in the Department of Dermatology, the First Affiliated Hospital of Kunming Medical University, Kunming, Yunnan, China. The research protocol was examined and approved by the ethic committee of Chinese Clinical trial Registry (ChiCTR) and was registered in ChiCTR (ChiCTR‐INR‐17012531). Benefits, risks, and potential complications were explained to the volunteers, and informed written consent was obtained from participants.

### 2.2 Study subjects

A total of 90 Chinese patients were recruited from the dermatology clinic of First Affiliated Hospital of Kunming Medical University. Diagnosis of melasma was made by dermatologists specialized in pigment disorders. Inclusion criteria included (a) males and females aged 25‐50 years old, without known systemic disorders; (b) willing to use SPF > 30 sunscreen during the entire study period without direct exposure to the sun; (c) not using any other freckle or whitening products or receiving other treatments (drugs, chemical stripping, laser, etc) at least 1 month before entering the study and during the whole period; (d) willing to sign the informed consent and to complete the study. Exclusion criteria included (a) pregnancy, breastfeeding, with systemic disorders (such as severe gynecologic, endocrine, tumors, and immunodeficiency diseases, etc); (b) having other skin diseases (herpes simplex, eczema, ulceration, active facial acne, etc); (c) currently attending other clinical studies or patients who have participated in other clinical studies within 3 months.

### 2.3 Test cream

This new whitening cream, manufactured by Beitaini Biotechnological Co., Ltd. (China), containing China camellia (1%), sanchi (0.5%), prinsepia utilis oil (0.5%), and portulaca oleracea (1%), which were added at low temperature (45℃) and mixed evenly at speed of 138 *g* for 5 minutes. The collection and extraction of these herbal ingredients were performed by State Key Laboratory of Phytochemistry and Plant Resources in West China, Kunming Institute of Botany, Chinese Academy of Sciences. A cream without active ingredients was used as placebo, and arbutin cream was used as additional control. The tested cream and the controls were certified by China Food and Drug Administration (CFDA) (G20170790; G20150147).

### 2.4 Treatments

Volunteers were randomly divided into three groups using a table of random numbers. Patients in group A were treated with a cream containing herbal mixture, while groups B and C were treated with arbutin cream and placebo, respectively, twice daily on the whole face for 12 weeks. Because ultraviolet light can aggravate pigmentation, all volunteers were instructed to apply the same sunscreen every 3 hours while being outdoor during the trial period.

### 2.5 Evaluation method

A Visia (Canfield Scientific, Inc, New York, NY, USA) was used to photograph the patients’ face at baseline and after 4, 8, and 12 weeks of treatments. The MASI score^22^ was used to evaluate the severity of disease independently by two individual observers. Because Mexameter^®^ is reliable and widely used for the objective assessment of pigmentation and erythema, melanin index (MI), and erythema index (EI) were measured using Mexameter® (MX 18; Courage & Khazaka, Germany). A mean value of three measurements per subject was taken at each time point.

Density of inflammatory cells was assessed with a reflectance confocal microscopy (RCM; Vivascope 1500; Lucid Inc, Rochester, NY, USA).^7^ To evaluate the density of inflammatory cells in melasma, each image (500 × 500 μm) was assessed and scored independently by two individual observers. In comparison to normal controls, the density was scored as 0 = no increase, 1 = slight increase, 2 = moderate increase, and 3 = marked increase.^23^ The score is presented as mean ± standard deviation. The higher the score was, the higher density of inflammatory cells was.

Safety, efficacy, and tolerability were evaluated at the end of week 4, week 8, and week 12. The volunteers were asked to evaluate their satisfaction with the following criteria: 0 = not satisfied, 1 = partially satisfied, 2 = satisfied, or 3 = very satisfied. We recorded adverse events, including itching, scaling, erythema, burning, and erosion at each visit.

### 2.6 Statistical analysis

The data were analyzed by SPSS version19.0 and were expressed as mean ± standard deviation. Repeated Measures ANOVA were used to compare data among three groups at different times. Statistical significance was assumed for a *P* < 0.05.

## 3 RESULTS

### 3.1 Demographic characteristics of patients

The patients were 40.35 ± 6.02 years old and had skin type III or IV (Fitzpatrick skin types). The mean duration of melasma was 5.46 ± 3.72 years. The baseline MASI scores were 12.83 ± 6.49 (detailed in Table 1).

**Table 1:** Baseline characteristics of subjects

Characteristics	Group A (N = 30)	Group B (N = 30)	Group C (N = 30)	Total (N = 90)
Age (y)	40.37 ± 7.22	40.4 ± 5.67	40.3 ± 5.18	40.35 ± 6.02
Fitzpatrick skin types				
III	14 (46.7%)	15 (50%)	14 (46.7%)	43 (47.8%)
IV	16 (53.3%)	15 (50%)	16 (53.3%)	47 (52.2%)
Baseline melasma score	13 ± 6.14	12.65 ± 8.04	12.84 ± 5.17	12.83 ± 6.49
Duration of melasma (y)	5.45 ± 3.53	5.47 ± 4.00	5.45 ± 3.74	5.46 ± 3.72

Data are presented as mean ± standard deviation.

### 3.2 The cream of herbal mixture improves MASI scores, MI, and EI

**Figure 1:**
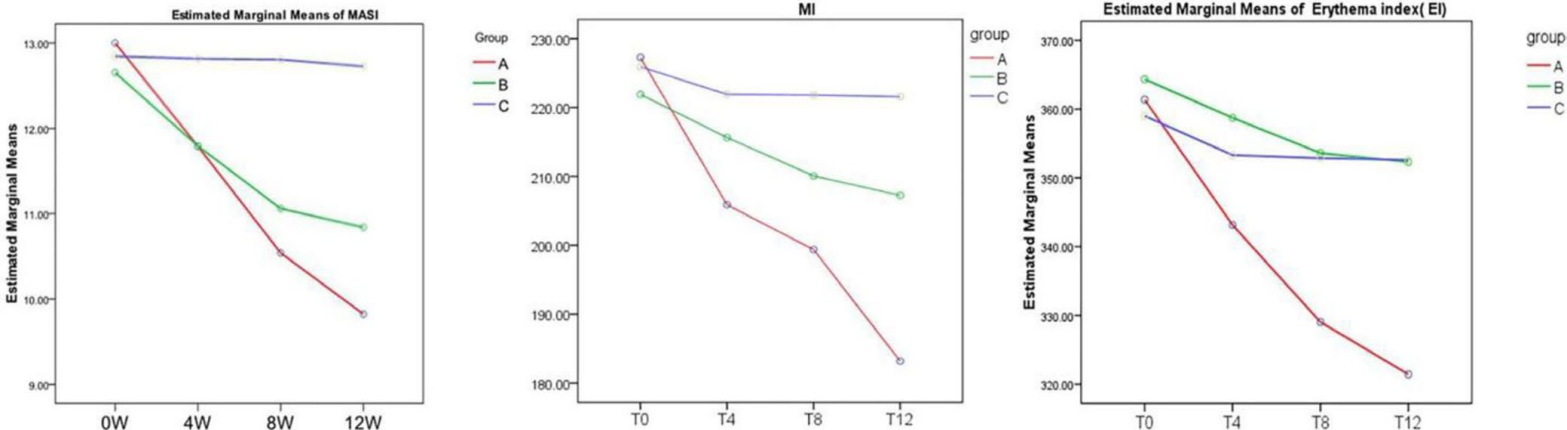
Changes in MASI score, MI (melanin index), EI (erythema index) after 12 weeks of treatment. Red line corresponds to test cream (A), green line to arbutin cream (B) and blue line to placebo cream (C)

As shown in Table 2 and Figure 1, after 12‐week treatments, both the cream of herbal mixture (A) and arbutin cream (B) significantly improved MASI scores (*P* < 0.05 vs baseline for both groups), whereas a more dramatic reduction in MASI scores was observed in group A (13.00−9.82 = 3.18 vs 12.65−10.84 = 1.81). Likewise, following 12‐week treatments, the average melanin index (MI) markedly decreased in both groups A and B in comparison with the baseline (Group A: 227.27−183.18 = 44.09; Group B: 221.9−207.23 = 14.67). Again, a more significant reduction in MI was observed in Group A than that in Group B (*P* < 0.05). However, only the test cream, but not arbutin cream, dramatically lowered erythema index following 12‐week treatments (361.37−321.43 = 39.94, *P* < 0.05 vs baseline). In contrast, 12‐week treatments with placebo did not significantly improve MASI scores and EI (12.84−12.73 = 0.11 for MASI scores; 359.0−352.6 = 6.4 for EI). These results indicate that the cream of herbal mixture improves melasma.

**Table 2:** Changes in MASI score, MI, EI, Inflammatory cells at week 0, week 4, week 8, and week 12

	Group A (N = 30)	Group B (N = 30)	Group C (N = 30)
MASI 0	13.00 ± 6.14	12.65 ± 8.04	12.84 ± 5.17
MASI 4	11.79 ± 5.34	11.79 ± 7.70	12.82 ± 5.19
MASI 8	10.54 ± 4.65	11.06 ± 6.80	12.81 ± 5.18
MASI 12	9.82 ± 4.43	10.84 ± 6.83	12.73 ± 5.19
MI 0	227.27 ± 76.42	221.90 ± 51.67	225.90 ± 51.22
MI 4	205.87 ± 46.83	215.63 ± 49.96	221.90 ± 49.69
MI 8	199.37 ± 37.47	210.07 ± 47.62	221.80 ± 50.86
MI 12	183.18 ± 44.19	207.23 ± 48.44	221.57 ± 51.27
EI 0	361.37 ± 39.27	364.33 ± 74.99	359.00 ± 58.37
EI 4	343.13 ± 50.25	358.73 ± 46.08	353.30 ± 52.41
EI 8	329.03 ± 54.82	353.60 ± 58.57	352.87 ± 48.56
EI 12	321.43 ± 51.10	352.30 ± 54.57	352.60 ± 55.38
Inflammatory cells at baseline	0.67 ± 0.66	0.60 ± 0.62	0.60 ± 0.62
Inflammatory cells at week 4	0.43 ± 0.63	0.57 ± 0.63	0.60 ± 0.62
Inflammatory cells at week 8	0.33 ± 0.61	0.57 ± 0.63	0.53 ± 0.57
Inflammatory cells at week 12	0.23 ± 0.50	0.53 ± 0.63	0.57 ± 0.63

Data are presented as mean ± standard deviation.

### 3.3 The cream of herbal mixture decreases inflammation

**Figure 2:**
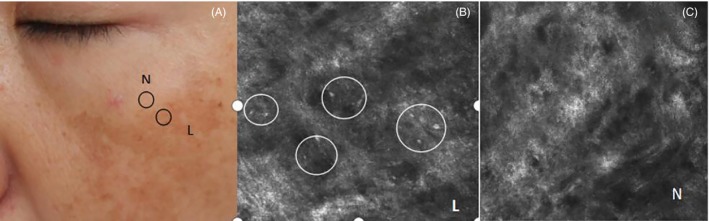
Melasma with Inflammatory cells. A, Clinical photograph of hyperpigmented macules (circled) on the cheek. The lesional (L) and perilesional normal skin (N) were evaluated. B, Confocal images show increased Inflammatory cells in the superficial dermis of the lesion (L) compared to perilesional normal skin (N) (C)

As seen in Figure 2, more inflammatory cells were observed in melasma in comparison with normal controls at baseline (Figure 2B vs C). Following 12‐week treatments with the cream of herbal mixture, the density of inflammatory cells markedly decreased (0.67 ± 0.66 at baseline vs 0.23 ± 0.50 after treatment, *P* < 0.05). In contrast, neither arbutin cream nor placebo significantly changed the density of inflammatory cells (Table 2). These results demonstrate that the cream of herbal mixture alleviates cutaneous inflammation in melasma.

### 3.4 Subjective satisfaction score

**Figure 3:**
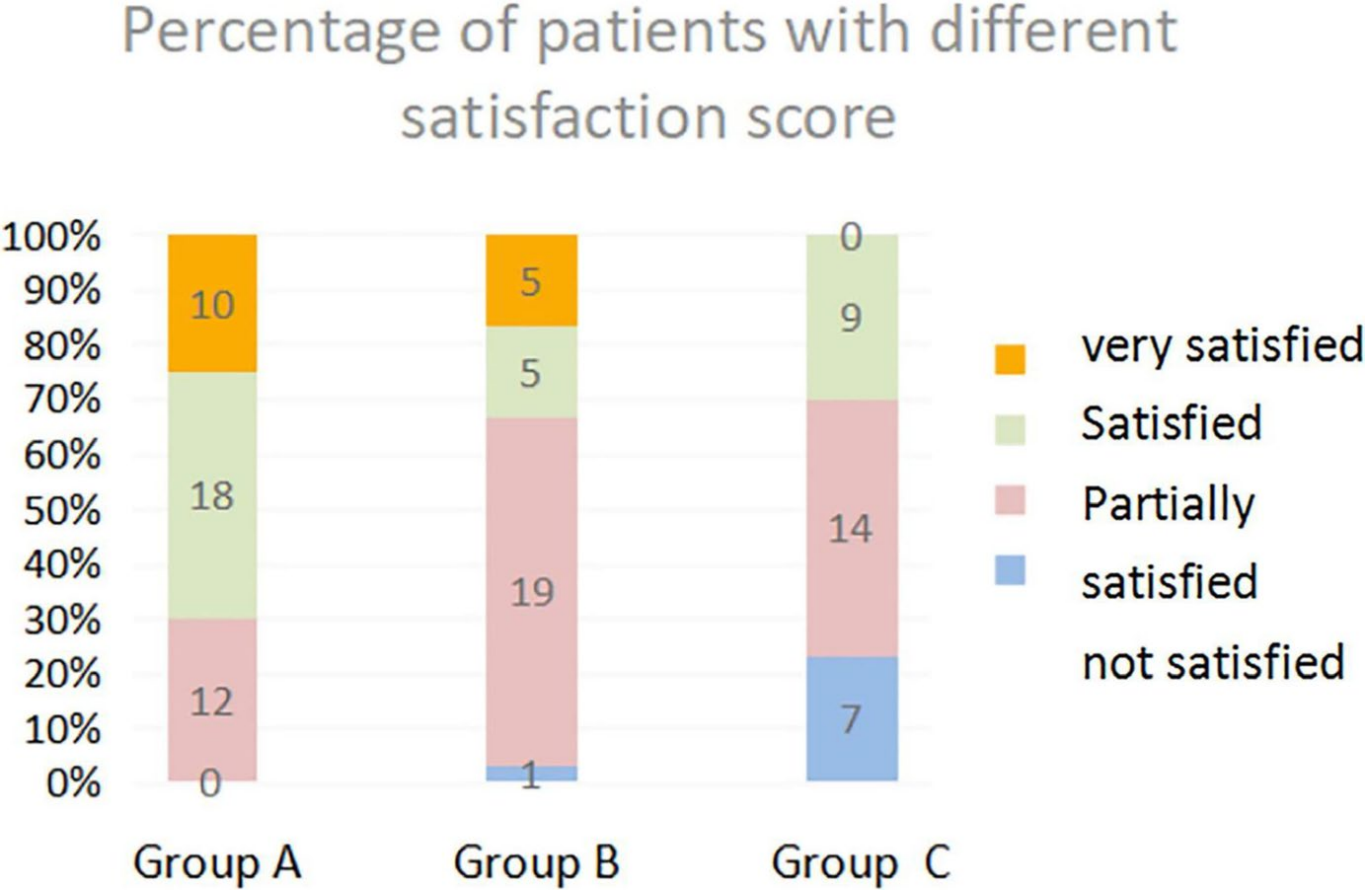
Percentage of patients with different satisfaction score for group A (test cream), group B (arbutin cream), and group C (placebo) at week 12

The subjective satisfaction scores were markedly improved in both group A and group B (Figure 3). In particular, the number of patients with “very satisfied” (score 3) increased from 4 (13.3%) at week 4 to 10 (33.3%) at week 12 in group A, while in group B, 2 patients (6.7%) at week 4 and 5 patients (16.7%) at week 12 had satisfaction score 3. In contrast, no patients felt “very satisfied” and 7 patients (23.3%) felt unsatisfied at week 12 after treatments with placebo.

### 3.5 Adverse reactions

No itching, scaling, erosion, burning, or ulcer was reported at any visit in group A and group C. Two subjects treated with arbutin cream experienced slight erythema and pruritus, which disappeared after stop using the arbutin cream.

## 4 DISCUSSION

**Figure 4:**
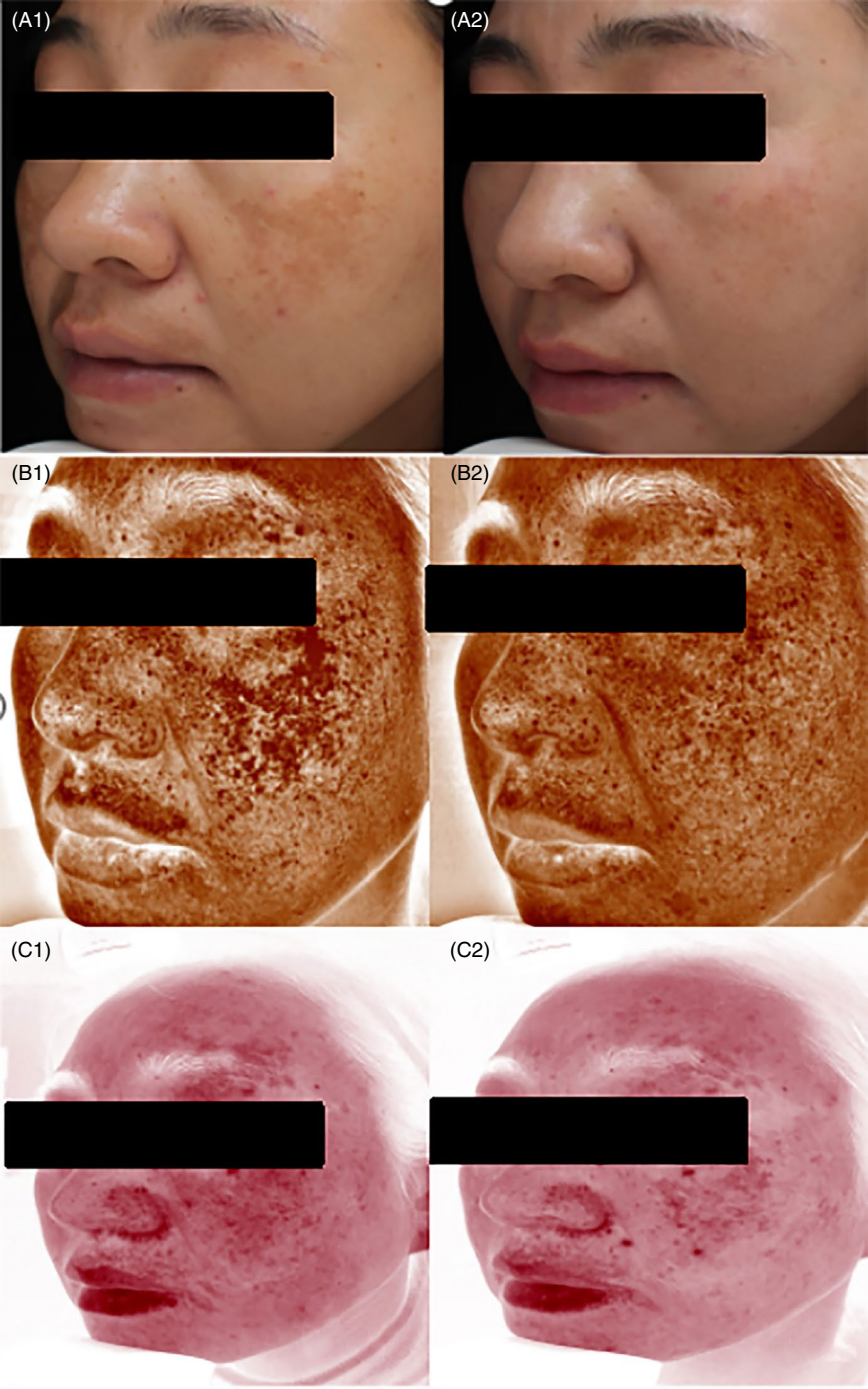
Representative images of study subjects of group A at baseline (A1, B1, and C1) and week 12 (A2, B2, and C2). A, Photographs were taken using natural light; B, browns spots. C, Clinical observations showing diminished red patch.

**Figure 5:**
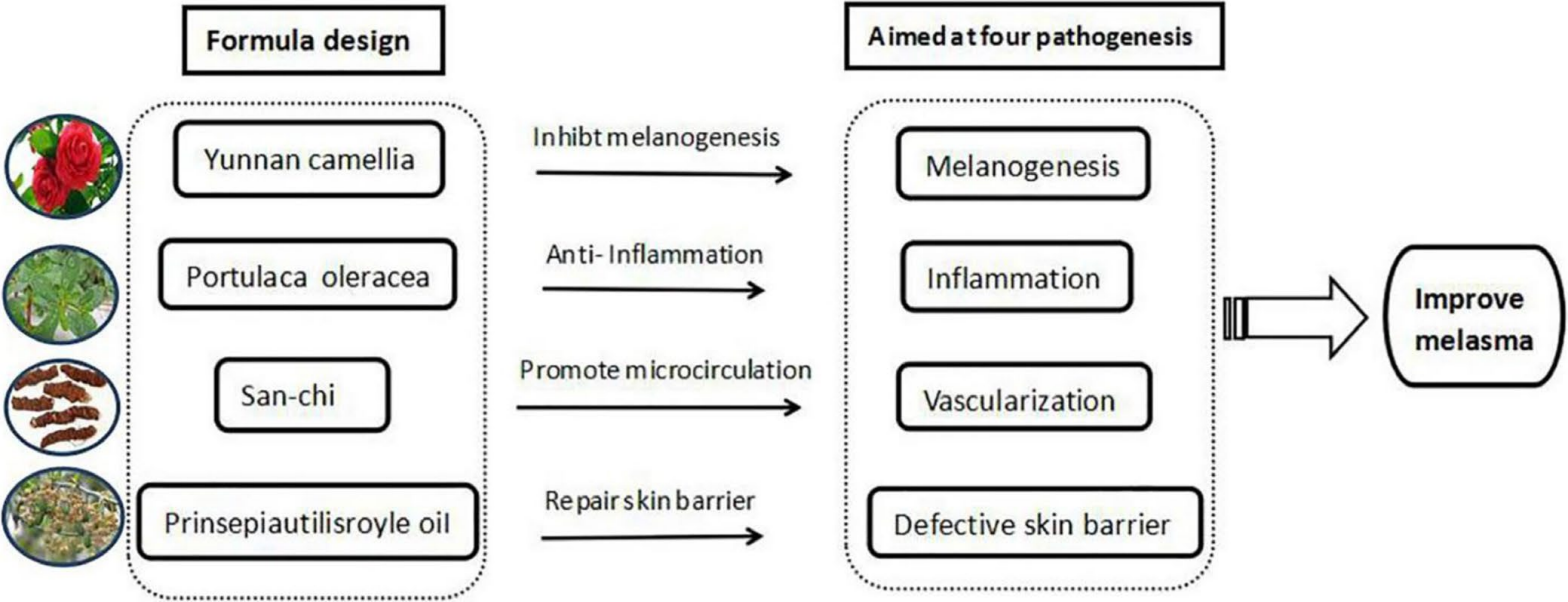
Formula design of the new whitening cream aimed at four main pathogenesis of melasma

Current regimens for melasma include chemical peels, oral drugs, topical therapy, prevention of UV radiation and laser therapies, most of which are easy to relapse and incomplete clearance.^24^ Because hyperpigmentation is the major clinical manifestation of melasma, much attention has been paid to decrease melanin by inhibition of the exacerbated activity of melanocytes and dispersion of melanin granules.^25^ Indeed, the strategies decreasing melanin can improve skin pigment to some extent. But recurrence is inevitable in most cases, if not in all cases. However, here we developed a cream containing herbal mixture, in which each natural ingredient specifically targets respective pathogenic aspect of melasma (Figures 4 and 5). Our results showed that topical applications of this cream containing herbal mixture markedly improved melasma.

Although we did not look at the molecular mechanisms, the pharmacologic mechanisms by which this herbal mixture cream improved melasma could be largely attributed to the four natural ingredients, that is, China camellia, sanchi, prinsepia utilis oil, and portulaca oleracea. Our previous studies demonstrated that extract of China camellia inhibited tyrosinase activity and proliferation of melanocytes.^18^ Moreover, camellianin A exhibits potent antioxidant activity,^26^, ^27^ while oxidative stress can cause hyperpigmentation.^28^ Thus, both antioxidant and inhibition of melanogenesis properties of China camellia can contribute to improvements in pigmentation in melasma following the treatments with the test cream.

Sanchi (Sanchi ginseng Panax notoginseng) is another active ingredient in our formulation. Previous studies have shown that Panax notoginseng saponins (PNS), a constituent of Sanchi, exhibited multiple health benefits, including improvements in microcirculation,^29^, ^30^ anti‐inflammation,^31^ and anti‐oxidation,^32^ while disturbed microcirculation, inflammation, and oxidative stress play pathogenic role in the development of melasma.^4^, ^7^, ^11^ Hence, it is likely that sanchi contributes to improvements in pigment, inflammation, and erythema index after 12‐week treatments with this cream of herbal mixture.

Our prior studies demonstrated that melasma is featured by infiltration of lymphocyte‐based inflammatory cells in the superficial dermis and capillaries and increased expression of Toll‐like receptors 2 and 4.^6^ Moreover, 75% of melasma patients displayed mild lymphocytic infiltration in the lesion.^7^ Furthermore, melasma patients with inflammatory infiltration had a more severe hyperpigmentation.^9^ In the present study, we demonstrated that topical treatments with the cream of herbal mixture decreased the density of inflammatory cells in melasma lesion. This benefit of the test cream could be due to the anti‐inflammatory and antioxidant properties of portulaca olerace in the cream, as demonstrated in prior studies.^33^, ^34^

Defective epidermal permeability barrier has be proposed to contribute to the development of melasma.^12^, ^13^, ^14^, ^15^ Accordingly, improvement in epidermal permeability barrier could benefit melasma. It has been demonstrated that Prinsepia utilis, an ingredient in the test cream, could improve epidermal permeability barrier via stimulation of epidermal lipid production, including ceramides, major component of lamellar lipids in the stratum corneum.^21^, ^35^ Thus, Prinsepia utilis induced improvement in epidermal permeability barrier could be additional mechanism by which the cream of herbal mixture improves melasma.

Taken together, the present study demonstrated that the cream containing herbal mixture improves melasma by multiple mechanism, including anti‐inflammation, antioxidant, improving microcirculation, inhibition of melanogenesis as well as improving in epidermal permeability barrier.

## 5 CONCLUSION

The cream containing herbal mixture is effective and safe to melasma.

## References

[1] Sanchez NP , Pathak MA , Sato S , Fitzpatrick TB , Sanchez JL , Mihm MC Jr . Melasma: a clinical, light microscopic, ultrastructural, and immunofluores‐cence study. J Am Acad Dermatol. 1981;4(6):698‐710. 6787100 10.1016/s0190-9622(81)70071-9

[2] Vachiramon V , Suchonwanit P , Thadanipon K . Melasma in men. J Cosmetic Dermatol. 2012;11(2):151‐157. 10.1111/j.1473-2165.2012.00613.x 22672280

[3] Rodrigues M , Pandya AG . Melasma: clinical diagnosis and management options. Australas J Dermatol. 2015;56(3):151‐163. 25754098 10.1111/ajd.12290

[4] Ogbechie‐Godec OA , Elbuluk N . Melasma: an up‐to‐date comprehensive review. Dermatol Ther (Heidelb). 2017;7(3):305‐318. 28726212 10.1007/s13555-017-0194-1 PMC5574745

[5] Kang Wh , Yoon Kh , Lee E‐s , et al. Melasma: histopathological characteristics in 56 Korean patients. Br J Dermatol. 2002;146(2):228‐237. 11903232 10.1046/j.0007-0963.2001.04556.x

[6] Wang YJ , Gu H , Guo MH , Tu Y , He L . Expressions of toll‐like receptors 2 and 4 in skin lesions and peripheral blood from patients with chloasma. Chin J Dermatol. 2015;48(2):100‐103.

[7] Kang HY , Bahadoran P , Suzuki I , et al. In vivo reflectance confocal microscopy detects pigmentary changes in melasma at a cellular level resolution. Exp Dermatol. 2010;19(8):e228‐e233. 20497220 10.1111/j.1600-0625.2009.01057.x

[8] Rodríguez‐Arámbula A , Torres‐Álvarez B , Cortés‐García D , Fuentes‐Ahumada C , Castanedo‐Cázares JP . CD4, IL‐17, and COX‐2 are associated with subclinical inflammation in malar melasma. Am J Dermatopathol. 2015;37(10):761‐766. 26381025 10.1097/DAD.0000000000000378

[9] Noh TK , Choi SJ , Chung BY , et al. Inflammatory features of melasma lesions in Asian skin. J Dermatol. 2014;41(9):788‐794. 25132344 10.1111/1346-8138.12573

[10] Kim EH , Kim YC , Lee ES , Kang HY . The vascular characteristics of melasma. J Dermatol Sci. 2007;46(2):111‐116. 17363223 10.1016/j.jdermsci.2007.01.009

[11] Passeron T . Melasma pathogenesis and influencing factors ‐ an overview of the latest research. J Eur Acad Dermatol Venereol. 2013;27(Suppl 1):5‐6. 23205539 10.1111/jdv.12049

[12] Wang YJ , Tu Y , Gu H , Humber P , He L . Explore the possible mechanism of epidermal permeability barrier dysfunction in melasma. China Med Abstr Dermatol. 2017;34(04):468‐472.

[13] Lee DJ , Lee J , Ha J , et al. Defective barrier function in melasma skin. J Eur Acad Dermatol Venereol. 2012;26(12):1533‐1537. 22077137 10.1111/j.1468-3083.2011.04337.x

[14] Kang HY , Suzuki I , Lee DJ , et al. Transcriptional profiling shows altered expression of wnt pathway‐ and lipid metabolism‐related genes as well as melanogenesis‐related genes in melasma. J Invest Dermatol. 2011;131(8):1692‐1700. 21562572 10.1038/jid.2011.109

[15] Torres‐Alvarez B , Mesa‐Garza IG , Castanedo‐Cazares JP , et al. Histochemical and immunohistochemical study in melasma: evidence of damage in the basal membrane. Am J Dermatopathol. 2011;33(3):291‐295. 21317614 10.1097/DAD.0b013e3181ef2d45

[16] Camarasa JG , Serra‐Baldrich E . Exogenous ochronosis with allergic contact dermatitis from hydroquinone. Contact Dermatitis. 1994;31(1):57‐58. 7924303 10.1111/j.1600-0536.1994.tb01914.x

[17] Kolbe L , Mann T , Gerwat W , et al. 4‐n‐butylresorcinol, a highly effective tyrosinase inhibitor for the topical treatment of hyperpigmentation. J Eur Acad Dermatol Venereol. 2013;27(Suppl 1):19‐23. 10.1111/jdv.12051 23205541

[18] Huang XF , Liu HY , He L . Effects of Camellia retic ulata, Chloranthus japonicas, Dodonaea viscosa and Paris polyphylla on the proliferation of and tyrosinase activity in a melanocyte cell line. Chin J Dermatol. 2015;48(2):133‐136.

[19] Wang Y , Chu Y , et al. Advances in study on saponins in manax notoginseng and their pharmacological activities. Chin Trad Herb Drug. 2015;46(09):1381‐1392.

[20] Pang Q , He L . Research in anti‐inflammation function by various extracted method and concentration of Portulaca oleracea. Dermatol Venereol. 2012;34:318‐322.

[21] Tu Y , Gu H , Li N , Pang Q , He L . Effects of Prinsepia utilis Royle oil on the synthesis of ceramide and expression of ceramidase. Chin J Dermatol. 2012;45:718‐722.

[22] Kimbrough‐Green CK , Griffiths CE , Finkel LJ , et al. Topical retinoic acid (Tretinoin) for melasma in black patients. Arch Dermatol. 1994;130(6):727‐733. 8002642

[23] Zhang QY , Sun DJ , Ying TU , et al. Clinical staging of melasma. Chin J Med Aesthet Cosmet. 2018;24(4):274‐278.

[24] Sarkar R , Chugh S , Garg VK . Newer and upcoming therapies for melasma. Indian J Dermatol Venereol Leprol. 2012;78(4):417‐428. 22772611 10.4103/0378-6323.98071

[25] Haddad AL , Matos LF , Brunstein F , Ferreira LM , Silva A , Costa D Jr . A clinical, prospective, randomized, double‐blind trial comparing skin whitening complex with hydroquinone vs. placebo in the treatment of melasma. Int J Dermatol. 2003;42(2):153‐156. 12709008 10.1046/j.1365-4362.2003.01621.x

[26] Piao MJ , Yoo ES , Koh YS , et al. Antioxidant effects of the ethanol extract from flower of Camellia japonica via scavenging of reactive oxygen species and induction of antioxidant enzymes. Int J Mol Sci. 2011;12(4):2618‐2630. 21731461 10.3390/ijms12042618 PMC3127137

[27] Liu Y , Luo X , Lan Z , et al. Ultrasonic‐assisted extraction and antioxidant capacities of flavonoids from Camellia fascicularis leaves. CyTA J Food. 2018;16(1):105‐112.

[28] Diehl C . Melanocytes and oxidative stress. J Pigment Disord. 2014;1:127.

[29] Wang J , Xu J , Zhong JB , et al. Effect of Radix notoginseng saponins on platelet activating molecule expression and aggregation in patients with blood hyperviscosity syndrome. Chin J Integr Med. 2004;24(4):312‐316. 15143716

[30] Yan QF , Tan PZ , Liu QT , et al. Influence of Panax notoginseng saponins on serums ICAM‐1 in patients with acute cerebral infarction. Chin J Pract Nerv Dis. 2008;1:56‐57.

[31] Yang BR , Yuen SC , Fan GY , Cong W‐H , Leung S‐W , Lee S‐Y . Identification of certain Panax species to be potential substitutes for Panax notoginseng in hemostatic treatments. Pharmacol Res. 2018;134:1‐15. 29772270 10.1016/j.phrs.2018.05.005

[32] Yang J , Yuan YZ , Wei , et al. Research progress of chemical composition and pharmacological actions of Panax notoginseng. Modern Trad Chin Med Materia Med World Sci Technol. 2017;19(10):1641‐1647.

[33] Zakaria M , Islam MW , Radhakrishnan R , Habibullah M , Chan K . Evaluation of anti‐inflammatory activity of Portulaca species. J Pharmacy Pharm. 2011;50:227‐231.

[34] Islam MW , Zakaria M , Radhakrishnan R , Habibullah M , Chan, . K. Evaluation of analgesic activity of the aerial parts of Portulaca oleracea v. sativa and its comparison with two related spices. J Pharmacy Pharm. 2011;50:226‐230.

[35] Hu L , Man H , Elias PM , Man M‐Q . Herbal medicines that benefit epidermal permeability barrier function. Dermatol Sin. 2015;33:90‐95.

